# Digitalised exercise material in forensic odontology

**DOI:** 10.1007/s00414-021-02740-7

**Published:** 2021-11-20

**Authors:** Ina C. Knivsberg, Simen E. Kopperud, Mai-Britt Bjørk, Gerald Torgersen, Katarzyna Skramstad, Sigrid I. Kvaal

**Affiliations:** 1 University of Oslo, Oslo, Norway; 2 Norwegian Armed Forces Joint Medical Services, Dental Division, Oslo, Norway

**Keywords:** Hybrid learning, Digitalised teaching, Intraoral scanning, Forensic education

## Abstract

**Introduction:**

This paper presents digital educational material in forensic odontology, including dental identification after multiple fatalities and dental age estimation from different age groups.

**Material and method:**

Electronic patient records consisting of intraoral scans of the dentition, digital radiographs, photographs and written dental records were collected. Exercises in age estimations contained digital radiographs and photographs of ground tooth sections, with digital measuring tools and tables according to age groups. The teaching material was organised as a module in an electronic Learning Management System with external links to all relevant teaching material.

**Results:**

For the identification exercises, intraoral scans and the latest digital radiographs simulated the *postmortem* examination of the deceased. For comparison, all other radiographs, photographs and dental records were available as *antemortem* material. The exercise was to match *postmortem* findings with the *antemortem* records using the Interpol standard and reconciliation. Age assessment of children used designated tables to grade tooth development on digital radiographs. For adults, non-destructive methods, digital radiographs, photographs and measuring tools were used.

**Discussion:**

The teaching concept was hybrid, but it can easily be adapted as a fully digital exercise. The instructions and written material can be translated into different languages. The level of difficulty in the exercises can be adjusted according to the participant’s level of knowledge.

**Conclusion:**

The educational material embraces the new possibilities for digitalisation and intraoral scanning. This might be a valuable tool for motivating and engaging the students in their participation and understanding of the subject.

## Introduction

Forensic odontology (FO) or forensic dentistry applies dental knowledge to criminal and civil laws enforced by police agencies in a criminal justice system. Investigative agencies may request assistance from a forensic odontologist to identify recovered human remains [1, 2]. General dental practitioners may be asked to assist the forensic odontologist. Many dental schools have included a general introduction to FO in the undergraduate curriculum [3–6].

Dental identification of human remains implies comparing dental records from a missing person with the deceased’s dental status. The dental record, generally a chronological series of treatment details, is transcribed to a status record, the *antemortem* (AM) record. Following the examination of the remains, the body’s dental status is registered as the *postmortem* (PM) record. Matching records AM and PM may provide dental identification [7]. Since 1984, Interpol has published and revised standards for human identification. It may be established by comparing AM and PM data collected from either fingerprints, dental examinations, DNA analyses and/or physical indicators [7].

**Fig. 1:**
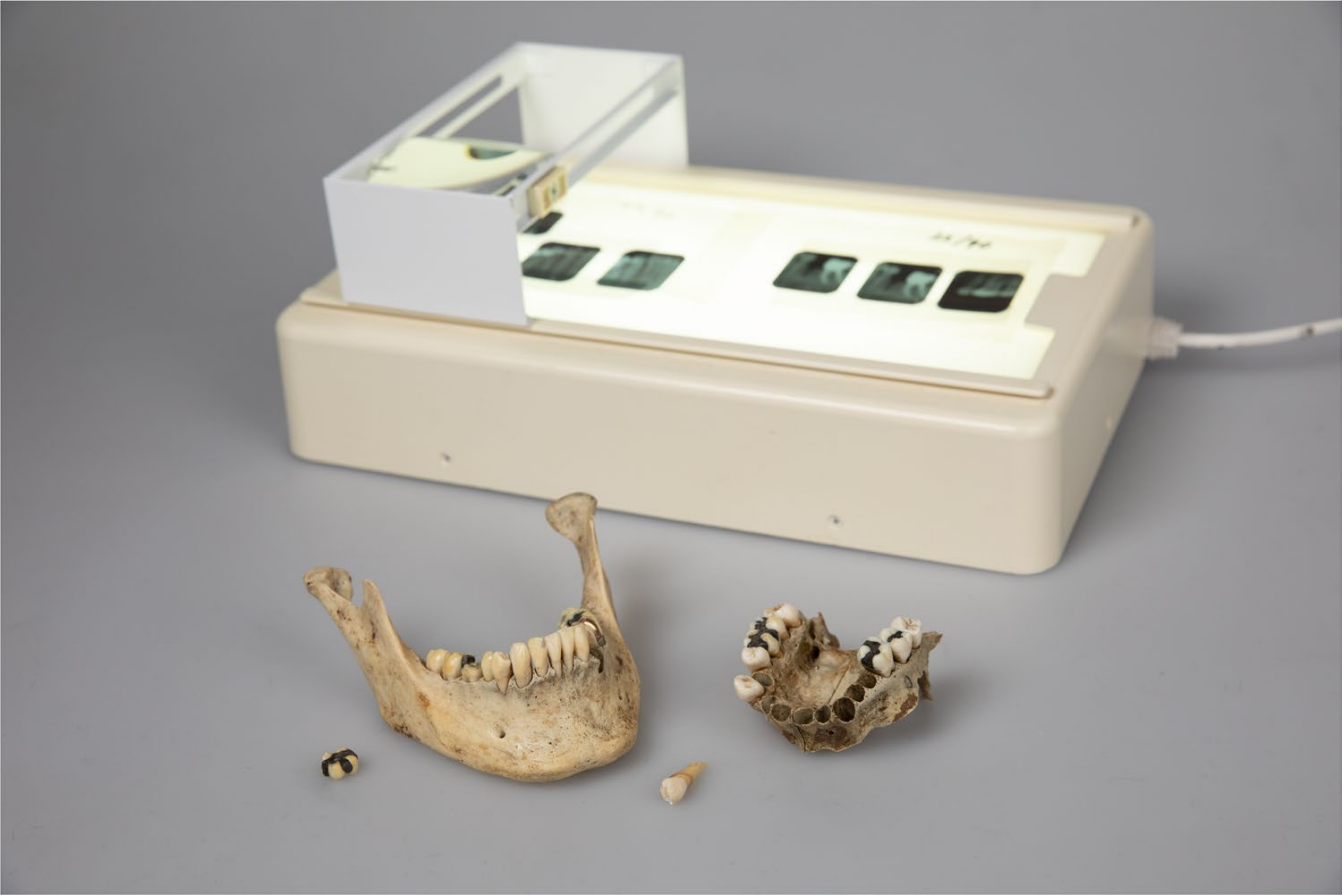
The educational material used prior to our new digitalised educational programme; lightbox with analogue intraoral radiographs and deteriorated material from human remains

Dental age estimation is based on dental development from foetal life to young adult [8]. In adults, dental age estimation is based on degenerative changes in the dentition [9]. For the practical part of the FO course, it has previously been recommended that jaws from anatomical dissections are used in addition to transcription or construction of dental records [10–12]. A disadvantage has been that the jaws must be returned to the body and new teaching material created every year. As an alternative, dry anatomical specimens have been used. However, with repeated handling, such material deteriorates (Fig. 1). The dental records and analogue radiographs will, over time, be outdated and may not comply with national regulations and recommendations for record-keeping.

The European General Data Protection Regulation (GDPR) was implemented on May 25, 2018, in all member states of the European Union (EU) and the European Economic Area (EEA) to harmonise data privacy laws [12]. GDPR and the Norwegian Act of applying biotechnology in human medicine (The Biotechnology Act 2003) raised the need to renew the teaching material providing identification and age estimation training.

These regulations paved the way for the digitalisation of older material and introduced the extended use of intraoral scanning (IOS).

IOS are either based on contact or non-contact between the object to be captured and the device to transfer information in direct optical impressions. Most scanners used in dentistry are non-contact type. Intraoral scanners use various methods to capture raw data, including confocal microscopy, triangulation, interferometry, wavefront sampling, structured light, laser and video [13]. The scanning software processes the images of the dento-gingival tissues using nonionizing electromagnetic radiation [14]. Digital information on oral structures collected by intraoral scanners can be managed through software tools to generate point clouds that are triangulated to give surface reconstructions, often referred to as meshes [15]. The software creates an STL file (standard tessellation language) and can be communicated to dental laboratories and patients. The scans can also be exported to a Polygon File Format file (PLY) which can be viewed in programmes on most platforms [16]. The 3D surface model is the ‘virtual’ alternative to the traditional plaster model, but it allows enhancement in magnification, rotation and colour adjustments [17].

### Aims

The purpose of this paper is to present digital educational material for practical exercises in FO.

Two types of exercises are included:

- dental identification after multiple fatalities
- dental age estimation from different age groups.

## Materials and methods

The new teaching concept used patient records from living patients for both AM and PM material. Electronic patient records were collected from private practices and a university dental clinic in Norway. Written consents were obtained from all participants; no remuneration was given. The patient records consisted of available radiographs, photographs and written dental records from the past 5 years. An intraoral scan (IOS) of the dentition, bitewings and intraoral and extraoral pictures substituted a clinical PM examination of the victim’s dentition and characteristic traits.

Using a standardised protocol, intraoral scans were performed with Primescan by Dentsply Sirona (2019) and 3Shape TRIOS 4 (2019). To avoid being dependent on specific software to view these scans, the files were converted to MPEG-4 format using the Adobe Media Encoder software (Adobe Inc. 2020) and Exocad Webview (exocad GmbH 2020). This enabled the students to access the 3D dentition scans as video clips on their computers. Dental records were converted to pdf files, radiographs anonymized using the software ImageJ and the pdfs anonymized with redact function in Adobe Acrobat DC (Adobe Inc. 2020) [18].

Digitalised and anonymized panoramic radiographs and periapical radiographs were used for the exercises in age assessment. For one of the exercises, an extracted tooth (premolar) was donated from a person of known age. The tooth was fixed in epoxy using EpofixResin/Epofix Hardener (Struers, Ballerup, Denmark), sectioned in the sagittal plane in a 1-mm slice using a Struers saw, and then photographed with a Single-Lens Reflex camera next to a millimetre scale. The free mathematics software GeoGebra Math Apps was used to measure given dimensions [19]. It provided intercalibrated measurement facilitating the completion of the digital formulas presented for age estimation.

The University of Oslo has adopted Canvas (Instructure, Inc.) as its standard Learning Management System (LMS). The FO’s teaching material was organised as a module in Canvas. It contained external links to all relevant teaching material, including a “user manual” for the assignments, handouts from FO lectures, and general information. The students accessed the material using their laptop computers. At the completion of the exercise, the students were asked to fill out a short evaluation form.

## Results

The implementation of the present teaching material was considered by the Regional Committee for Medical Research Ethics in Norway not to require approval (ID: 2019/460 D).

### Included patient records (sample)

The patient records from 15 females and 16 males had an age span ranging from 12 to 92 years of age. The two groups of collected patient records consisted of regular recall patients from private clinics and referred patients to the specialist clinic at the University. This ensured widespread treatment modalities, sex, age and different record-keeping software, and provided a significant variation in identification markers. For the five age assessment exercises, panoramic and periapical radiographs were anonymized and transferred from analogue to digital format. A photograph visualised apical translucency.

### The practical exercise in identification

**Fig. 2:**
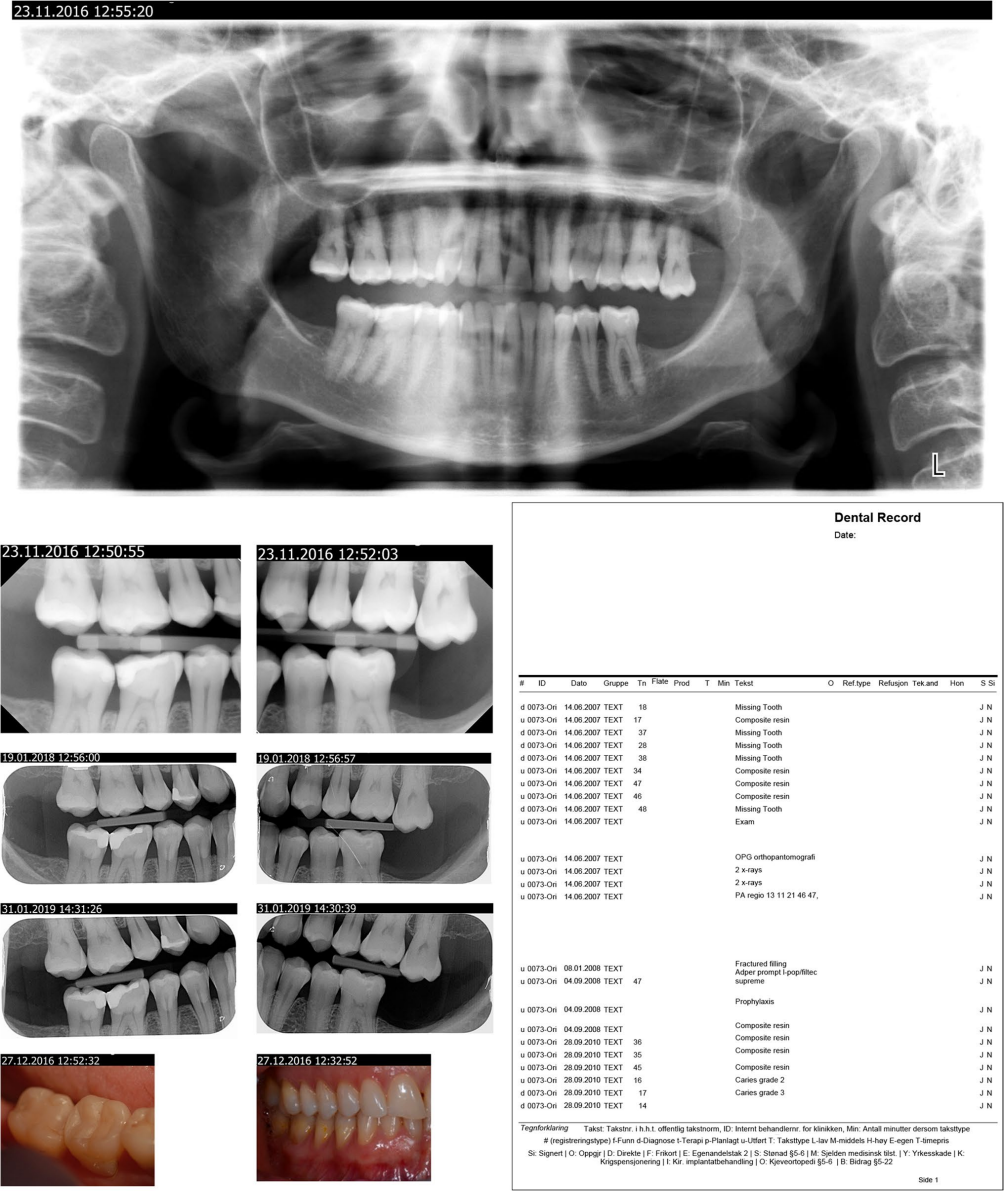

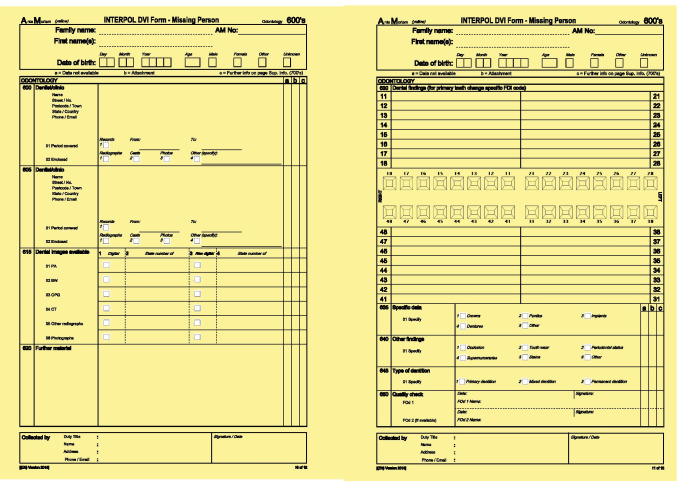
**a** AM information package as presented for the students; intraoral pictures, bitewings radiographs, panoramic radiographs, written dental records. **b** Interpol form, disaster victim identification, missing person antemortem.

**Fig. 3:**
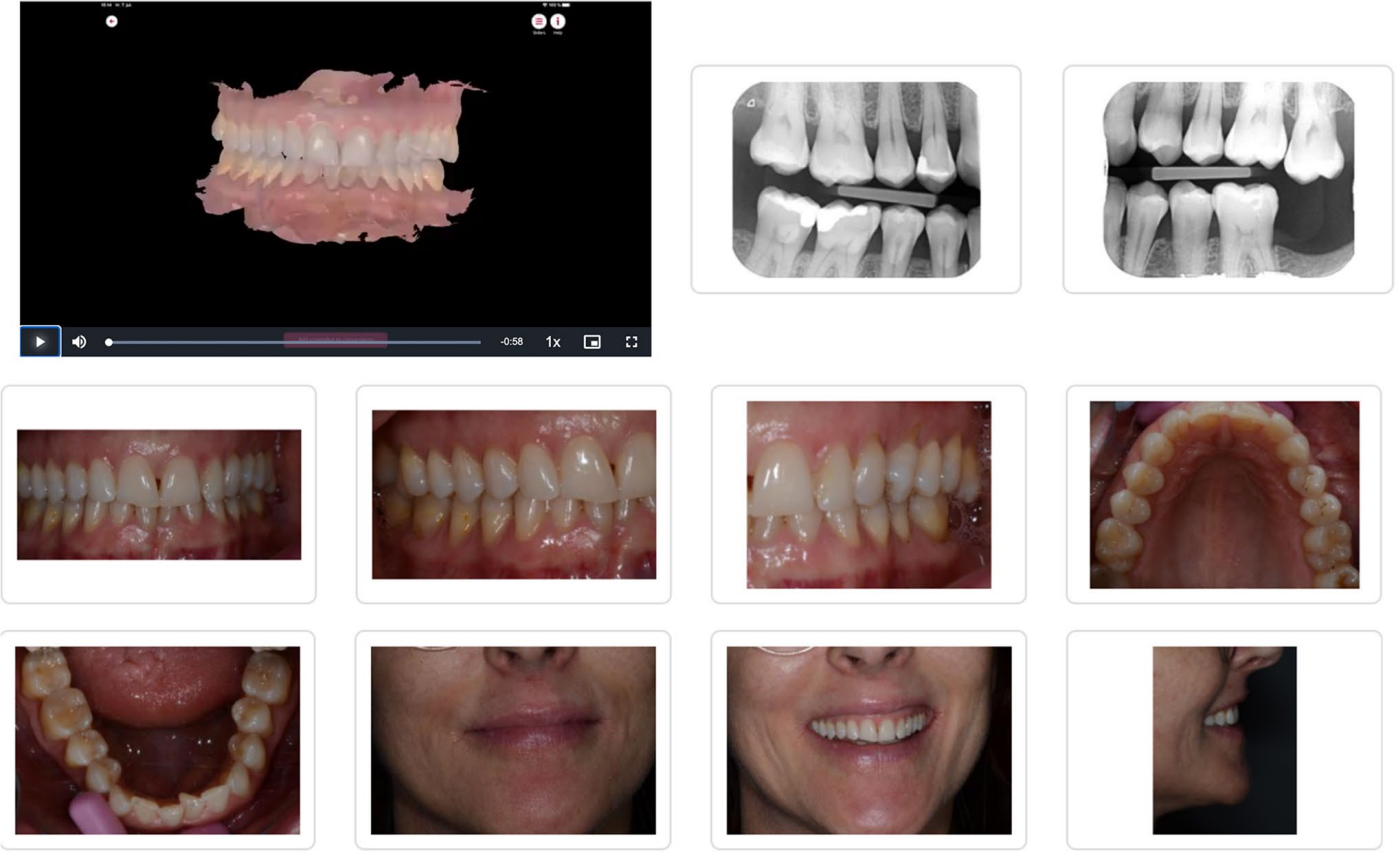

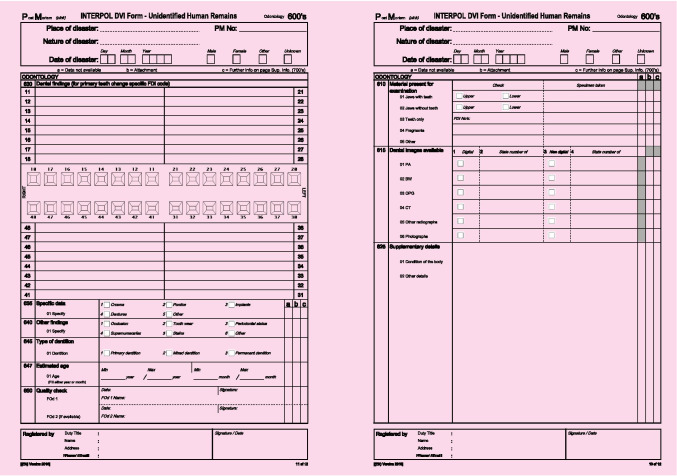
**a** PM information package as presented for the students; newly performed bitewing radiographs, intraoral pictures, video clips of the IOS scan. **b** Interpol form, disaster victim identification, unidentified human remains postmortem.

Each patient record was split into two separate patient information packages, providing 31 AM packages and 31 corresponding PM packages. The PM packages consisted of the video clip of the intra-oral scan of the dentition, along with the most recent radiographs and oral photographs, resembling the information a forensic dentist would gather during a postmortem examination (Fig. 3a). The AM packages consisted of all other radiographs, photographs and dental records, resembling information that could have been collected from a dental practitioner (Fig. 2a). The AM and PM packages were randomised in a list with a cross-reference security key.

**Fig. 4:**
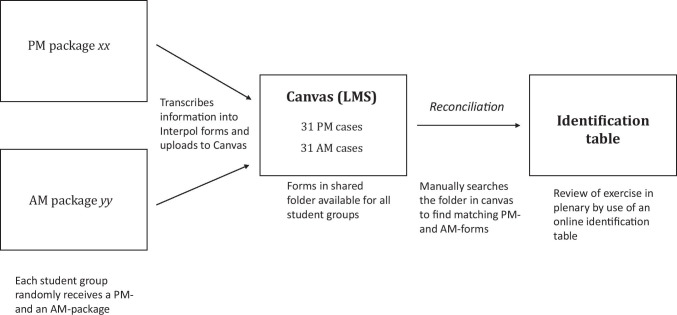
A simplified flow diagram of the exercise in identification

The practical exercise simulated an accident with multiple fatalities. The students’ task in the practical exercise was to reconcile a PM case correctly with a matching AM case after downloading editable pdf versions of Interpol’s AM and PM forms [7] (Figs. 2b and 3b). Each student received one PM information package and transcribed the information to Interpol’s PM form after going through its content. They uploaded the PM form to a shared folder in Canvas. All students then randomly received an AM information package and transcribed the information to Interpol’s AM form. Finally, they manually searched the shared folder with PM forms until they found a dentition matching their AM case. They filled in a reconciliation form, uploaded the form to a shared folder in Canvas, and finally, the exercise was reviewed in plenary by use of an identification table (Fig. 4).

### Practical exercise in age assessment

The practical exercise in age assessment consisted of five separate assignments with specific digitalized information packages. This practical exercise aimed to demonstrate some of the principles for age assessment in different age groups, as presented in Table 1.

**Table 1:** Methods for age-assessment

Age group	Material	Method
Children	Panoramic radiographs	Demerijan, London Atlas ([20] [21])
Sub-adults	Panoramic radiographs	Lee, Haavikko ([22, 23])
Adults	Periapical radiograph of upper left central incisor	Kvaal ([24])
Dead	Ground section of lower premolar	Bang ([25])

Specific methods were suggested for the different age groups. Until the age of 12–14, several teeth are developing, and the students had to evaluate results from two different methods [20, 21]. When the second molar is fully developed at the age of 16, the third molar is the only tooth still developing. Two different tables for assessment of wisdom teeth development in relation to age were provided [22, 23].

For the assessment of adults, both a *non-destructive* and a *destructive* method were chosen. The non-destructive method included measurements of the pulp size on radiographs since a reduction in pulp size can be seen with advancing age [24]. The destructive method used a photograph of a sectioned premolar. An increase in the apical translucent zone can be measured on teeth with advancing age. This method is often used in identification and age estimations of human remains (Bang’s method) [25].

The students were asked to perform the age assessments and enter their answers into the LMS (Canvas). Their assessments were immediately indicated as right or wrong within a reasonable age span using a numerical range in the Canvas Quizzes function. Each assignment was discussed before the students advanced to the next exercise.

### Background story

To make both practical exercises more realistic, a fictional story was presented for the students. The accident was a wedding celebration in a cave where all participants were poisoned by carbon monoxide from a generator due to poor ventilation—an incident that ended fatally for some of them. The students formed an identification team that was asked to identify the deceased and estimate the age for some of the victims.

### Implementation of the practical exercises

Both practical exercises started with a 45-min introductory lecture, repeating the most relevant theoretical principles needed to perform the exercises. The lecturers explained the purpose of each exercise. The students worked in pairs using their laptops, solved each exercise and uploaded their answers into Canvas. Teachers in FO were available for assistance while the students worked with the exercises. When all tasks were completed and registered, the exercises were reviewed in a plenary session to ensure the students’ highest cognitive skills in educational taxonomy [26].

### Evaluation

Fifty-five dental students attended the practical exercises. The response rates of a questionnaire on students’ satisfaction with the practical exercises were 80% and 73% for identification and age estimation respectively. The students generally rated all aspects of the new teaching methods as high. Some students indicated that they had software compatibility problems when using other operating systems than Windows. Some general comments on digital teaching were submitted.

## Discussion

The present paper describes the digitalisation and implementation of practical exercises on dental identification and age assessment. The introduction of FO to dental students is essential as dentists may serve a critical humanitarian function by supplying local communities with forensic resources [27]. FO teaching in the undergraduate curriculum is best suited to the final year when the students’ knowledge in dental morphology, pathology, radiology and clinical dentistry has evolved. The exercises allow students to consolidate their learning outcomes. Teaching of FO varies significantly among dental schools around the world. The lack of teaching resources or differences in the offered training in FO may introduce inequalities between dentists.

It is complicated to bring a class into a mortuary for ethical, legal and practical reasons. Restrictions in the number of students allowed to be in the morgue, in the number of bodies available for identification purposes, and restrictions related to the confidentiality of the diseased hamper such a teaching method. Even small, selected groups may disrupt routines and permission for a visit may not be granted.

Ideally, all students should have the opportunity to perform a dental PM examination in a morgue representing a realistic approach to forensic sciences. Important traits in the dentition may only be detectable by physical examination like probing, and this aspect cannot fully be represented by a digital method. Nevertheless, the combination of intra-oral scanning presented as a film, intra-oral pictures and radiographs will in most cases reveal even dental restorations without radio-opacity. The digital material does not represent a perfect solution, but is close enough to reality for undergraduate dental students.

Digital material like the one presented in this paper can be used in any LMS and reduces the need for human resources. The difficulty level in matching material can also be adjusted according to the participants’ level of knowledge. The material can be translated and expanded to be used in postgraduate courses.

The undergraduate FO course curriculum in dentistry may also include an introduction to forensic medicine, crime investigation, legal aspects and research, including practical experience in dental identification and dental age estimation [3]. A brief introduction to other forensic disciplines and how FO links with the wider forensic community may be included. The students get an impression of a dental PM examination and the importance of identification for the deceased’s family and society.

### Identification

Active participation in forensic odontology is for the selected few, but the concept and the understanding of the principle are essential for any practicing dentist. This enables the dentist to explain to relatives of the deceased the need for handing over the dental records to the police or other investigative agency. Likewise, the practitioner will understand the need for the submission of all recorded material. The late timing in the curriculum will also cement the understanding of why it is essential to keep accurate dental records and the importance of well-written records [27].

### Dental age assessments

Any dental practitioner may be asked to assess the development of a child’s teeth using available charts. These will give a rough estimate of normal development and may be sufficient to calm an anxious parent. In legal and other contexts such as sports, a more accurate assessment may be required. The students must understand the possibilities and limitations of different methods of age assessment, the biological variations in tooth development and the implications in legal matters.

Age assessments are multi-professional topics and open for cooperation between different disciplines. The variation in tooth development timing may be a question raised. The dentist ought to know how dental development can be assessed relative to chronological age for people without birth documents. Deciding whether he or she is a minor or an adult is relevant to the rights related to age in western societies.

Age estimations have been at the centre of controversies because there is no consensus on how such estimations should be performed and interpretated. The biological variation in age assessment is demonstrated in the exercises [28–30]. Evaluation of age assessment methods has been the topic of master theses, and the course may be an introduction to PhD research projects.

### Intraoral scans

Many general dental practitioners and prosthodontists use IOS as a diagnostic tool. It provides a virtual impression of the dentition and sometimes the whole oral cavity. Digital scanners are classified as medical equipment [31]. In our practical exercises, we used a 3D model constructed from a complete digital scan of the dentition as a model for PM examination. To our knowledge, the use of IOS is a new approach to visualise PM for educational purposes. It serves its purpose perfectly by being minimally time-consuming and neither exposing the patients to ionising radiation nor a complicated procedure. IOS has been shown to provide higher patient comfort than regular alginate impressions, but still maintaining a similar degree of accuracy [32, 33].

Making a film of the 3D scanning does not fully represent a PM examination. It cannot replace all the information a trained forensic odontologist can draw from a corpse, but serves as an introductory tool. An additional aspect in 3D scanning, not evaluated in the present study, is the capturing of the soft-tissue topography and morphological information, such as the palatal rugae. Traits like these can have high power in deduction related to sexual dimorphism and forensic identification [34, 35].

### Digital teaching

Blended or hybrid learning was implemented in these practical exercises. Blended learning entails integrating face-to-face interaction and technologically mediated interaction between students, teachers and learning resources [36, 37]. This is in line with Manica et al.’s statements that teaching forensic odontology has gone from blackboard to webinars [38]. Face-to-face lectures and technology may be combined with blended learning and virtual classrooms. Better accessibility, affordability, and more flexibility are some of the benefits of hybrid learning [39]. Digital access may increase students’ learning potential since all students could be actively involved.

With fully digital teaching, the students can learn anytime and anywhere, thereby developing new skills leading to life-long learning. It has been stated that students are more available mentally in a lecture theatre [40]. The evaluation confirmed that the students find the educational material in FO applicable in a fully digital platform, but simultaneously appreciate the teachers’ presence. It has also been confirmed in other evaluations that dental students viewed online learning as a supplement rather than a replacement of traditional teaching methods [41].

Combining the sectioning of a tooth with digitalised measurement tools and scanning analogue panoramic radiographs presented some challenges. Different computer-monitor sizes, resolutions and other qualities impaired diagnostic accuracy and results. The ambient light varies for the different workplaces of the students. Those are well-known challenges when working with digital radiographs [42, 43]. A standardised test may be considered to calibrate monitor settings to avoid image presentation inconsistencies on different devices. Such a test tool is provided by the American Association of Physicists in Medicine [44].

A consequence of the Covid 19 pandemic was an immediate transition to digital education in all theoretical parts of the dental subjects. Digital education is an essential tool to maintain social distance in education and take advantage of asynchronous learning [45]. It cannot replace the clinical part of dental education, which is crucial when educating a practitioner, but it can complement clinical teaching.

### Future perspectives

Augmented reality in 3D visualisation could be a prospect for dental education, enhancing insight in tooth morphology, position, occlusion and facilitating treatment planning [31]. Digital technologies also include the 3D printing of virtual teeth, which provides transparency for all students due to identical setups. This can be used to explain biomimetic dentistry, important in conservative dentistry and prosthodontics, but, as shown in this paper, could also be a valuable tool in FO identification cases.

Implementing new information or updating the database is easy. The level of difficulty may be adjusted by adding or removing cases. The material can easily be transformed into webinars. To fulfil an international application and enhance inter-university collaboration, the exercises may, in time, be translated into English, contributing to the education of international forensic odontologists.

## Conclusion

The digital educational material in FO embraces new possibilities for digital teaching, intraoral scanning and new strategies ensuring patient anonymity. In addition, it preserves the copyright of the authors. It provides a flexible learning environment and allows all the students to take a more active part in the exercises.

The material can be implemented in any LMS and any video conference platform. It is applicable for both under- and postgraduate students as a blended/hybrid or fully online course. The digitalisation of smaller topics like FO, which have unequal teaching resources in different dental schools, opens the possibility of providing more students the required knowledge without excessive expenses.

## Abbreviations

3D: three-dimensional
AM: antemortem
EU: European Union
EEA: European Economic Area
FO: forensic odontology
GDPR: General Data Protection Regulation
IOS: intra oral scans
LMS: learning management system
PAN: panoramic radiographs
PM: postmortem
PDF: portable document format
